# Vitamin D: Is it a primary hormone targeting the migraine headache or just as adjunct therapy?

**DOI:** 10.17712/nsj.2017.1.20160561

**Published:** 2017-01

**Authors:** Marwan S. Al-Nimer

**Affiliations:** *College of Medicine Al-Mustansiriya University Baghdad, Iraq College of Pharmacy Hawler Medical University Erbil, Iraq*

To the Editor

With great interest, I have read the review article entitled “New advances in prevention of migraine”, by Khalid W. Al-Quliti and Ekhlas S. Assaedi published in the Neurosciences.^1^ I would like to mention in this correspondence the value of prescribing vitamin D to migraine patients.

There is no doubt that vitamin D supplementation as a hormone or as nutraceuticals has a place in the management of chronic pain, including headaches. In one randomized, double-blind, placebo-controlled study, vitamin D supplementation (10 µg or 25 µg daily for 16 weeks) has been improving the headache score (using Visual Analogue Scale and Headache Impact Test -6) insignificantly but it did not show significant effect on the occurrence, anatomical localization, or degree of pain or headache versus placebo.^2^ The mechanism of vitamin D in pain symptoms depends greatly upon the type of pain involved and it relieves the pain of skeleton-muscular origin and headaches, possibly due to its reducing effect on the sensitivity of nerve fibers in the muscles. In patients with chronic unexplained pain, vitamin D supplementation improved the quality of life and reduced the pain score.^3^ Buettner et al^4^ reported the beneficial effect of combined therapy of vitamin D (1,000 international unit capsules twice daily) and simvastatin (20 mg tablets twice daily) up to 24 weeks, in reducing the number of migraine days in patients with episodic migraine.

In one cross section study that carried on 5938 patients aged >40 years old in the United State, it has been found that statins (lipid lowering agents) significantly reduced the severity of migraine headache when the patients have high serum levels (>57 nmol/L) of 25-hydroxy calciferol.^5^ In another study, vitamin D supplementation (50000 international unit per week for 10 weeks) significantly reduced the headache frequency in migraine patients aged 10-61 years old.^6^ It seems that the effects of vitamin D supplementation is not related to vitamin D deficiency. Mottaghi et al^7^ found a weak non-significant correlation between serum vitamin D and migraine severity, while a weak positive relationship with headache diary results were observed.

From these recent studies, vitamin D can serve as a primary drug targeting the migraine or as an adjunct therapy with other drugs that exert pleotropic effects as with statins.

## Reply from the Author

No reply received from the author.
